# Multipath TCP-Based IoT Communication Evaluation: From the Perspective of Multipath Management with Machine Learning

**DOI:** 10.3390/s20226573

**Published:** 2020-11-18

**Authors:** Ruiwen Ji, Yuanlong Cao, Xiaotian Fan, Yirui Jiang, Gang Lei, Yong Ma

**Affiliations:** 1 School of Software, Jiangxi Normal University, Nanchang 330022, China; jiruiwen@jxnu.edu.cn (R.J.); yiruijiang512@126.com (Y.J.); leigang@jxnu.edu.cn (G.L.); 2 Department of Computer Science and Engineering, University of Bologna, 40126 Bologna BO, Italy; xiaotian.fan@studio.unibo.it; 3 School of Computer and Information Engineering, Jiangxi Normal University, Nanchang 330022, China; may@jxnu.edu.cn

**Keywords:** IoT communication, multipath TCP, path management, machine learning

## Abstract

With the development of wireless networking technology, current Internet-of-Things (IoT) devices are equipped with multiple network access interfaces. Multipath TCP (MPTCP) technology can improve the throughput of data transmission. However, traditional MPTCP path management may cause problems such as data confusion and even buffer blockage, which severely reduces transmission performance. This research introduces machine learning algorithms into MPTCP path management, and proposes an automatic learning selection path mechanism based on MPTCP (ALPS-MPTCP), which can adaptively select some high-quality paths and transmit data at the same time. This paper designs a simulation experiment that compares the performance of four machine learning algorithms in judging path quality. The experimental results show that, considering the running time and accuracy, the random forest algorithm has the best performance in judging path quality.

## 1. Introduction

With the extensive application of the Internet-of-Things (IoT) network technology and users’ increasing interest in various applications of IoT, the IoT traffic volume has increased significantly in the global Internet traffic [1,2]. At the same time, the development of various wireless access technologies (such as Wi-Fi, WiMax, LTE, etc.) has promoted modern IoT devices to be equipped with multiple network interfaces and attached with multiple heterogeneous access functions [3]. These devices can meet the data transmission requirements in the IoT environment through multiple network links, and are supported by the emerging multipath Transmission Control Protocol (MPTCP) technology [4].

**Figure 1:**
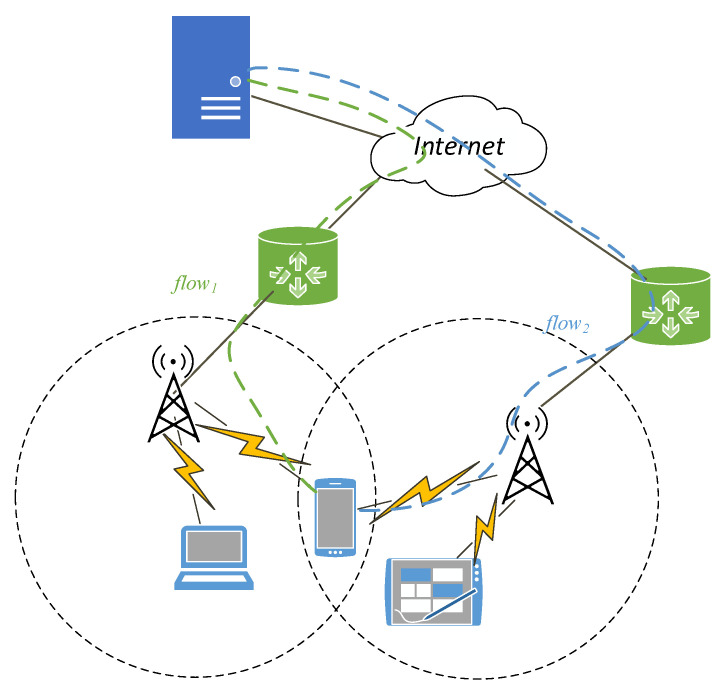
Schematic diagram of MPTCP transmission process.

Intelligent multi-homed devices can simultaneously schedule application data through multiple independent end-to-end available paths under the support of MPTCP, so as to achieve bandwidth aggregation, load balancing and dynamic switching, and automatically convert data from the most crowded and easily interrupted path to the better quality path [5]. Many studies have shown that concurrent multipath transmission based on MPTCP can effectively improve throughput performance and Quality of Service (QoS) [6,7]. Figure 1 is a schematic of the MPTCP transport process.

MPTCP has many advantages when applied to concurrent transmission of heterogeneous networks, but with the rapid development of the IoT, more and more devices are connected to the Internet, leading to greater data exchange. In complex real-world environments, cyber attacks are very common. The IoT environment is particularly vulnerable to distributed denial of service (DDoS) attacks, which further brings privacy risks [8,9]. Frequent changes in network quality cause network connections to have a negative impact on MPTCP performance, such as out-of-order arrival of data packets and buffer congestion, especially for time-sensitive multimedia streaming services [10,11,12,13]. The traditional transmission control mechanism based on static mathematical model can no longer meet the complexity and accuracy requirements of the future IoT. In order to make full use of the advantages of new communication technologies, researchers have carried out in-depth researches on the application of machine learning technology, the proposal of new transport protocols, privacy protection and the introduction of multipath functions at the transport layer. This shows that it is necessary to study the transmission control protocols and algorithms with intelligent learning and dynamic adaptive functions to automatically determine multipath quality and effectively manage multipath quality.

This paper presents an automatic learning path selection mechanism based on MPTCP. This mechanism can manage multiple paths based on decisions calculated from machine learning models. In this simulation experiment, we embed datasets with different delay and packet loss rates into a well-trained algorithm model to evaluate the performance of different machine learning algorithms in judging multipath quality. We hope to select the most accurate and efficient machine learning algorithm through research, and design a more intelligent path management scheme that can be applied to the actual environment according to the research results. The research results of this paper can provide new research ideas for related fields and help scholars to design a more optimized MPTCP path management scheme.

The rest of this article is organized as follows. The second part briefly introduces the relevant background of MPTCP path management. The third part introduces in detail our proposed MPTCP-based automatic learning path selection mechanism and four classic machine learning algorithms. The fourth part carries out simulation experiment design and performance evaluation. We discussed related issues in the fifth part, and the final part summarizes the article and prospects for future work.

## 2. Background and Related Work

MPTCP fundamentally changes the data scheduling and transmission mode, which effectively improves the transmission capacity and stability of the network. The path management function is to detect and use multiple paths between two hosts. When MPTCP is used for concurrent transmission over heterogeneous networks, the most important thing is how to effectively manage and utilize multiple asymmetric paths to maximize system throughput performance. In the current Concurrent Multipath Transport (CMT) solution, both the transport and retransmission strategies provided are relatively simple [14]. The standard MPTCP scheduler splits packets in a uniform manner across all available paths. In MPTCP connections, multiple paths will affect each other [15,16]. When the quality difference between the multipath is large, some unnecessary retransmissions will be frequently started and passed through the poor performance path, which may cause data confusion or even buffer blockage, and further seriously reduce the transmission performance [17,18,19]. Therefore, the lack of intelligent path management will cause various problems in the current MPTCP.

The network status changes in real time, and the current network status is always lagging based on the feedback information. MPTCP multipath management mechanism and broadband fitting algorithm based on traditional static mathematical model are difficult to meet the complexity and accuracy requirements. In order to solve this problem, researchers in this field have tried some research to design an intelligent path management scheme that can effectively control the use of paths. Y. Lim et al. [20] proposed cross-layer path management, which is based on link layer state control path usage. According to the state, the data transfer on the connected path is suspended and the path is released. B. Hesmans et al. [21] proposed a control plane containing the path management function to manage the use of different paths. R. K. Chaturvedi et al. [22] proposed a new MPTCP Adaptive Efficient Packet Scheduler (AEPS), which can utilize the bandwidth of all available paths to provide high throughput with the minimum completion time. Different from previous studies, J. Chung et al. [23] adopted a machine learning mechanism to control the use of paths, and proposed a new path management scheme called MPTCP-ML. The results show that MPTCP-ML is superior to traditional MPTCP in detecting path quality. D. A. F. Saraiva et al. [8] provided solutions for privacy protection and data protection in the context of Internet of Things. W. Li et al. [24] proposed a learn-based multipath congestion control method called SmartCC, which can significantly improve the total throughput and is superior to the latest mechanism in various performance indicators. Z. Xu et al. [25] have proposed a control framework based on deep Reinforcement Learning (DRL), which is superior to some famous MPTCP congestion control algorithms in throughput, without sacrificing fairness. Through investigation, M. Polese et al. [26] identified three major research trends related to transport protocols: (i) Application of machine learning technology; (ii) Development of new transport protocols; (iii) The introduction of multipath functions.

**Figure 2:**
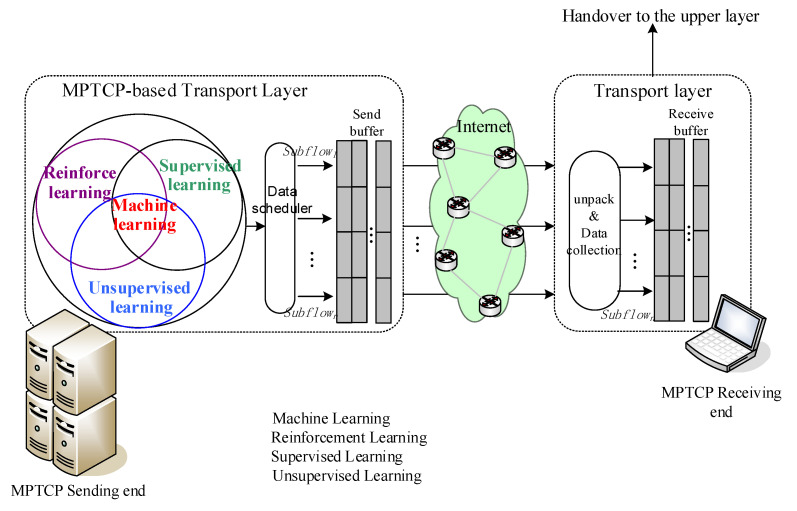
MPTCP multi-path transmission control mechanism incorporating machine learning methods.

Machine learning (ML) is an interdisciplinary subject. Figure 2 is a schematic of MPTCP multipath transport combined with machine learning. Machine learning algorithms are generally considered to be divided into unsupervised learning, supervised learning and reinforcement learning. We will thoroughly analyze the characteristics of several typical machine learning algorithms and use machine learning methods to learn and measure the transmission parameters of the path. By establishing a path quality evaluation model, MPTCP senders can predict the transmission quality of each path as quickly and accurately as possible. The machine learning method is an ideal alternative to the traditional static mathematical model, because it can optimize the robustness of multipath transmission systems and improve the quality of data transmission.

On the basis of previous researches, this paper further studies the path management scheme combining MPTCP and machine learning. Through simulation experiments, we compare the performance of different machine learning algorithms in judging the path quality, and select the optimal algorithm to design the multipath management scheme according to the performance evaluation results.

## 3. Design Overview of ALPS-MPTCP

Automatic learning path selection mechanism based on MPTCP (ALPS-MPTCP) uses machine learning algorithms. This mechanism has intelligent learning capabilities, which can optimize the robustness of the multipath transmission system, and achieve the purpose of reducing data out of sequence and avoiding blockage in the receiving buffer. Like MPTCP, ALPS-MPTCP is located below the application layer and above the TCP layer, and can provide a standard TCP interface for the application layer. The ALPS-MPTCP system consists of three parts: signal strength detector, path quality sampler and ML computing center. The signal strength detector performs signal strength detection tasks before the mobile device accesses the network. After the device is connected to the network, the path quality sampler collects the characteristic data of path quality every 100 milliseconds and inputs it to the ML computing center. The ML computing center receives the data (such as delay, packet loss rate, bandwidth) obtained from the sampler, and then uses intelligent computing to predict the path quality and perform effective path management. It determines the path to be used based on the quality of each data transmission path. If the path performance is poor, MPTCP-ML will suspend data communication on the path instead of keeping it. Repeat the above process to continuously determine the quality of each path. In addition, ALPS-MPTCP can be responsible for path management, data packet scheduling, sub-flow interface and congestion control. By using the data scheduler, the data flow can always go through the best path. Eventually, the data will reach the MPTCP receiving end through the Internet, stored in the receiving buffer and handed over to the upper layer. In short, these functions cooperate with each other. A multipath management system based on machine learning will obtain the best available path between two hosts. The design principles and optimization goals of ALPS-MPTCP can be summarized in five points: improving throughput, fairness, balancing congestion, security and resilience.

We used four classic and easy-to-implement machine learning algorithms (*k*-NN algorithm, random forest algorithm, *k*-Means algorithm, and reinforcement learning algorithm) to deal with the problem of MPTCP performance degradation. Finding the most suitable path selection algorithm is a key part of parallel multipath transmission. Different machine learning algorithms [27,28,29,30,31,32,33] may have their own advantages for specific network environments, but the network environment is constantly changing. We need to find the best-performing machine learning algorithm under different network environments and different service requirements, such as ensuring the real-time performance of data transmission, and the stability and accuracy of data transmission.

### 3.1. *k*-Nearest Neighbor Algorithm(*k*-NN)

*k*-NN [27] is a typical supervised learning algorithm. It is one of the most widely used algorithms in pattern evaluation. In supervised learning, each example is a pair of input objects (usually a vector) and an expected output value (also called a supervised signal). Supervised learning algorithms can analyze training data and generate an inference function that can be used to map new examples. The best solution would allow the algorithm to correctly identify class tags when the tags are not visible. *k*-NN is simple and easy to implement, but when the sample is unbalanced, the prediction bias will become large.

In the *k*-NN algorithm, *k* represents the number of nearest neighbors that help predict the test sample category. As for distance measurement, standard Euclidean distance is commonly used in *k*-NN case to measure the distances between the training set samples and test set samples. The standard Euclidean distance is defined below as [27]:

(1) $$d\left(x_{i},x_{y}\right)=\sqrt{\sum {\left(a_{r}\left(x_{i}\right)-a_{r}\left(x_{j}\right)\right)}^{2}}.$$

Similarly, *k*-NN calculates the most common categories of the *k* nearest neighbors to estimate the categories of test instances in the test set. Defined as the following Equation (2) [27]:

(2) $$c\left(x\right)=\underset{c\notin C}{\arg\;\max\;}\sum_{i=1\;to\;k}\delta (c,c\left(y_{i}\right)),$$

where $\left(y_{1},y_{2},y_{3},\cdots ,y_{k}\right)$ are the *k* nearest neighbors of a specific test instance of the test dataset, *k* is the number of the neighbors, *C* represents a limited set of class labels.

### 3.2. Random Forest Algorithm

**Figure 3:**
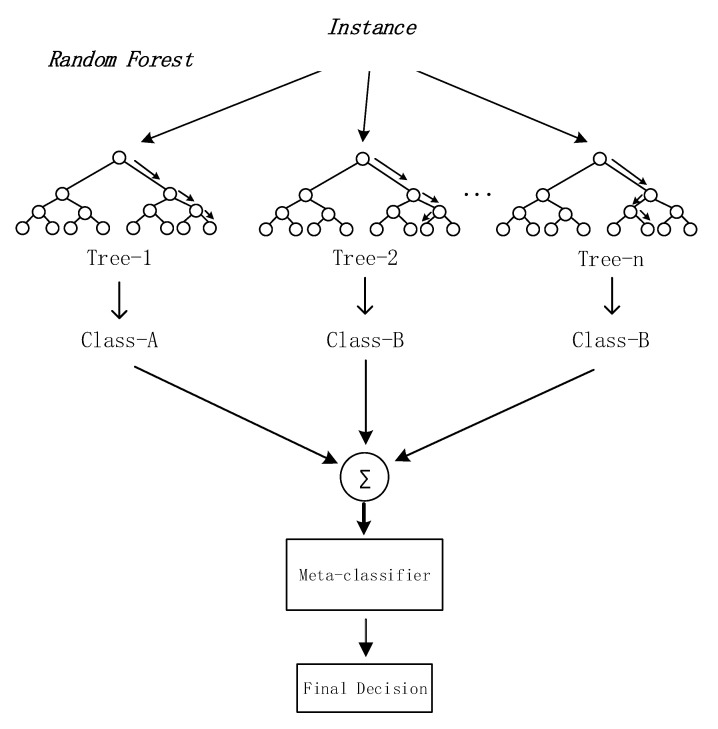
The workflow of Random Forest algorithm.

Random forest has fast training speed and strong generalization ability [28]. Random forest extracts multiple samples from the total sample set, builds a sub-dataset through a boot program, and then trains the features in the sub-dataset to form a basic decision tree (Repeating the above two steps to form a random forest). It can produce high-precision classifiers, but it cannot provide continuous output. Figure 3 is a schematic diagram of the working mechanism of the random forest algorithm [28]. Random forest contains multiple decision trees trained by Bagging ensemble learning technology. When the sample to be classified is input, the final classification result is determined by the output result of a single decision tree. Random forest solves the problem of decision tree performance bottleneck, has good tolerance to noise and outliers, and has good scalability and parallelism for high-dimensional data classification problems. Due to its good performance, the random forest algorithm has been widely used in bioinformatics, finance, computer vision, speech recognition, data mining and other fields.

First, we assume the given dataset as $D=\{x_{i},y_{i})$, $x_{i}\in R^{k}$, $y_{i}\in \left\{1,2,\cdots ,c\right\}$, the random forest as an *M* decision trees $\left\{g\left(D,\theta_{m}\right),m=1,2,\cdots ,M\right\}$. A combined classifier will be made up after a full process of learning. The classification result of the random forest output is determined by the major votes of the classification result of each decision tree [28].

### 3.3. *k*-Means Clustering Algorithm(*k*-Means)

*k*-Means algorithm is a classic unsupervised learning algorithm, which classifies the entire object. The *k*-Means algorithm is a representative of the typical prototype-based objective function clustering method [29]. It is the distance from the data point to the prototype as the objective function of optimization, and the adjustment rule of iterative operation is obtained through the method of function extreme value evaluation. The advantage of this algorithm is that it can handle large datasets, thereby maintaining scalability and efficiency, but it can only be used when the average value of the cluster can be defined, which may not be suitable for some applications.

For a given dataset containing n-dimensional data points $X=\{x_{1},x_{2},\cdots ,x_{i},\cdots ,x_{n}\}$, where $x_{i}\in R^{d}$ and the number of data subsets to be generated *k*, *k*-Means algorithm organizes the data into *k* partition $N=\{n_{k},i=1,2,\cdots ,k\}$. Each partition represents a category $c_{k}$, and each category $c_{k}$ has a category center $\mu_{i}$. The *k*-Means algorithm selects the Euclidean distance as the similarity and distance judgment criterion, and calculates the sum of the squares of the distances from the points in the class to the cluster center $\mu_{i}$ [29].

(3) $$J\left(n_{k}\right)=\sum_{x_{i}\in n_{k}}{\left|\right|x_{i}-\mu_{k}||}^{2}.$$

The goal of clustering is to minimize the sum of squares of total distances $J\left(N\right)=\sum_{k=1}^{k}J(n_{k})$.

(4) $$J\left(N\right)=\sum_{k=1}^{k}J(n_{k})=\sum_{k=1}^{k}\sum_{x_{i}\in n_{k}}{\left|\right|x_{i}-\mu_{k}||}^{2}=\sum_{k=1}^{k}\sum_{i=1}^{n}d_{ki}{\left|\right|x_{i}-\mu_{k}||}^{2},$$

subject to $d_{kn}=\left\{\begin{array}{c} 1,\;\;\mathrm{if}\;x_{i}\in n_{i}\\ 0,\;\mathrm{if}\;x_{i}\notin n_{j} \end{array}\right.$.

Obviously, according to the Least Squares method and the Lagrange principle, the cluster center should be taken as the average of the data points of the category class.

The *k*-Means algorithm begins with an initial *k*-category division and then assign each data point to each category to reduce the square of the total distance [29]. Because the total squared sum of distances in the *k*-Means clustering algorithm tends to decrease as the number of categories *k* increases (when k=n, J(N)=0). Therefore, the sum of squares of the total distance can only obtain the minimum value under a certain number of categories *k*.

### 3.4. Reinforcement Learning Algorithm

Reinforcement learning is having Agent to study in the form of “trial and error”, using the reward and environment interacting to guide behavior [30]. The goal of the agent is to collect the biggest reward. As an important machine learning method, Reinforcement Learning adopts the “attempt and failure” mechanism in human and animal learning, emphasizes learning in interaction with the environment, and uses evaluative feedback signals to optimize decision-making. The advantage of this algorithm is using reward functions to make seemingly random function behavior manageable, but the reward function is difficult to design.

Basic reinforcement is modelled as a Markov decision process. At each time *t*, the agent receives the state *s* and a reward *r* from the environment. The agent then have to selects and executes an action *a* from the set of available actions based on the state *s* and its experience of choosing action to optimize long-term reward. As it’s a long-term problem, the consequence of its actions is quite of importance in order to acting optimal even though the immediate reward might be negative.

Reinforcement learning algorithms can be divided into value function-based and strategy-based. In reinforcement learning based on value functions, the most commonly used learning algorithm is Q learning algorithm, and its iterative formula is as follows [30]:

(5) $$Q\left(s_{t},a_{t}\right)\leftarrow Q\left(s_{t},a_{t}\right)+\alpha \left[r_{t+!}+r\max_{a}Q\left(s_{t+!},a\right)-Q\left(s_{t},a_{t}\right)\right],$$

where $Q(s_{t},a_{t})$ is the state-action value at time *t*, the reward value is *r*, and α is the discount factor.

## 4. Performance Evaluation

### 4.1. Experimental Setup

The main equipment of our experiments includes the following:

(i) A wired server, located in JXNU (Jiangxi Normal University), the operating system is Fedora Core 6, the kernel version is 2.6.15. The server is connected to the JXNU network through the Ethernet interface;
(ii) Two mobile clients, that is, two Android smartphones as the client of the Skype voice call. We have introduced machine learning into MPTCP path management at the application layer, taking advantage of portability and convenience of access to a variety of information from wireless networks and mobile devices. The pre-built random forest algorithm, reinforcement learning algorithm, *k*-Means algorithm, and *k*-NN algorithm are embedded in the measurement application. In the simulation experiment, we used the characteristic parameters of LTE and Wi-Fi networks provided by the International Telecommunication Union (ITU), including fixed broadband values and interval values of delay and packet loss rate [31]. In order to ensure the fairness of the experiment, a wireless routing node was set up on each mobile device, and the two wireless routers used the same bandwidth. We generated random numbers within the parameter range to simulate various path environments.

### 4.2. Performance under the *k*-NN Algorithm

**Figure 4:**
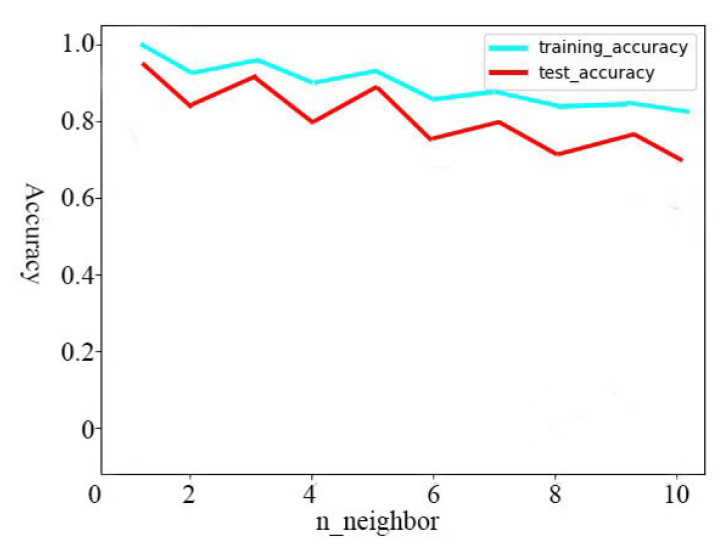
Different N_neighbors under Different Accuracy.

Figure 4 presents when the number of neighbors is 3, the accuracy of data results is the best, so we select 3 as the number of neighbors in the *k*-NN algorithm designed. As can be seen from Figure 4, the accuracy of *k*-NN algorithm in the training set is higher which average is 0.95, no matter how many neighbors there are, but the accuracy in the test set is higher when the number of neighbors is 3. When the number of neighbors increases, the accuracy decreases rapidly. So here we choose 3 as the neighbor number parameter. Then, we fit the classifier using the training set.

**Figure 5:**
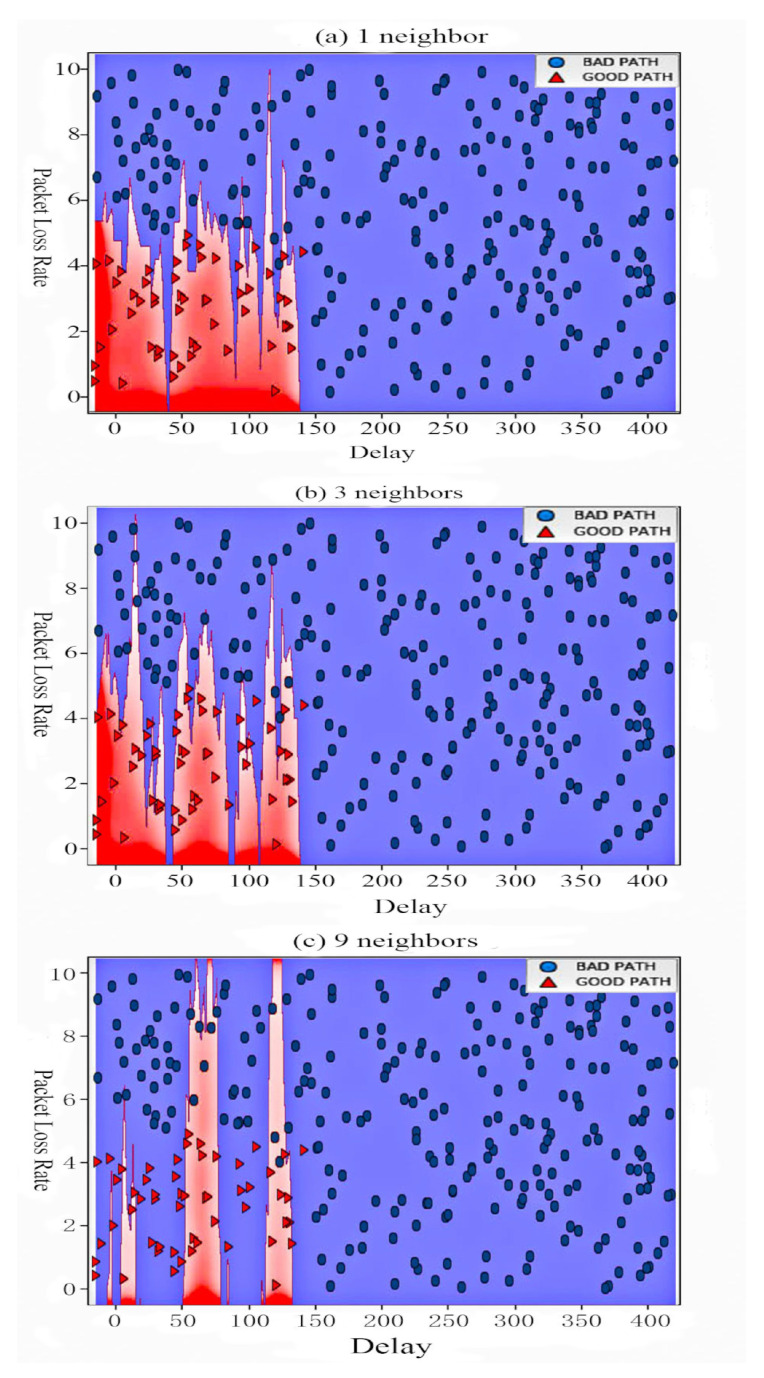
Performance under *k*-NN Algorithm.

Figure 5 shows the results of putting datasets with different delay and packet loss rates into the *K*-NN algorithm model we trained. It can be seen that the *k*-NN algorithm has better performance in distinguishing datasets with different delay and packet loss rates and judging path quality. To assess the degree of generalization of our *k*-NN model, we used a scoring method with test data and test label [32]. We see that our *k*-NN model has an accuracy of about 88%, which means that the model correctly predicts the class for the 88% sample in the test dataset.

### 4.3. Performance under the Random Forest Algorithm

**Figure 6:**
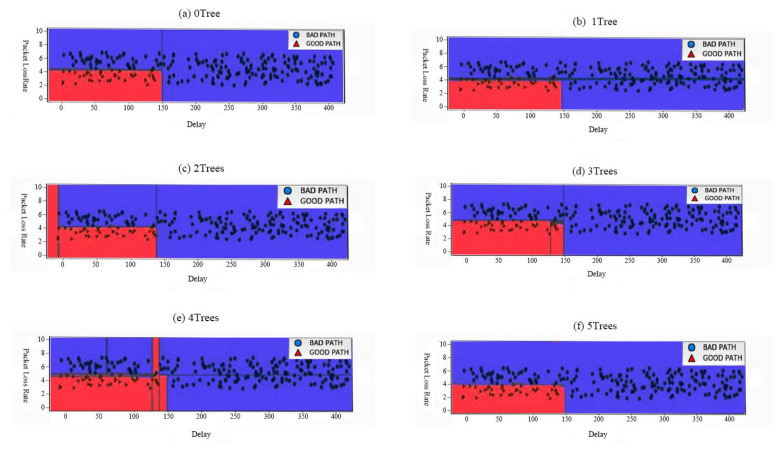
Performance with (**a**) 0 Tree, (**b**) 1 Tree, (**c**) 2 Trees, (**d**) 3 Trees, (**e**) 4 Trees, and (**f**) 5 Trees, respectively.

Figure 6 shows the application of the random forest algorithm. By increasing the number of trees, we compare the performance under different packet loss rates and delays. The red part is the good path, the blue part is the bad path. The figure shows their distribution. We can clearly see that the five tree learning decision boundaries are very different. Each of them made some mistakes because some of the training points drawn here were not actually included in the tree’s training set due to bootstrap sampling.

Figure 6 also shows the result, the random forest overfits less than any of the trees individually and provides a much more intuitive decision boundary. In the real application, we use many more trees (often hundreds or thousands), leading to even smoother boundaries. From Figure 6, we can see that random forest algorithm has a better performance in distinguishing data groups with different delay and packet loss rate, and it can better distinguish good path from the bad path. To evaluate how well our random forest model generalizes, we call the scoring method with test data together with the test labels [33]. We see that our random forest model is about 97% accuracy, meaning the model predicted the class correctly for 97% of the samples in the test dataset.

The random forest gives us an accuracy of 97%, better than the linear models or the single decision tree, without tuning any parameters. We also can see by the experimental results, the random forest model performance on the training set is nearly 100% accuracy.

### 4.4. Performance under the *k*-Means Algorithms

**Figure 7:**
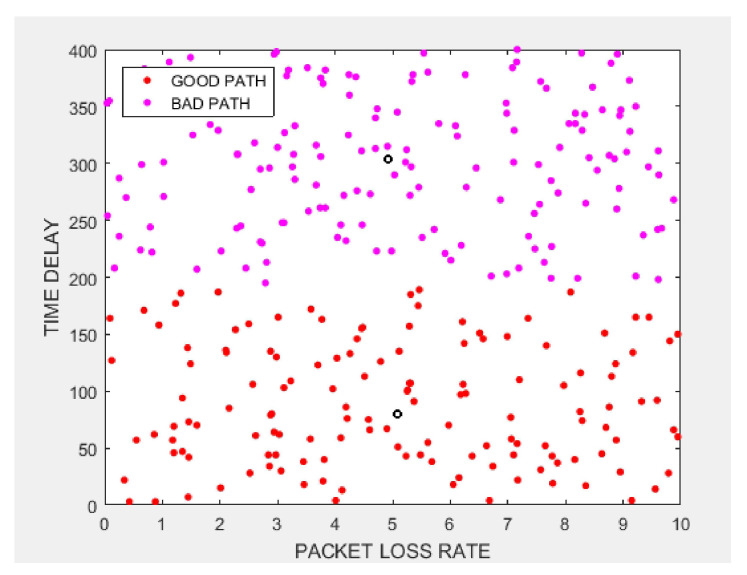
Performance under *K*-Means Algorithm.

Given new data points, *k*-Means algorithm model will assign each to the closest cluster center. Figure 7 shows the analysis of the packet loss rate and delay under the k-Means algorithm. The red part is the good path and the purple part is the bad path. From Figure 7, we use marked dataset to compare with the prediction results of *k*-Means model to obtain the accuracy of *k*-Means algorithm. We see that our *k*-Means model is about 72% accuracy, meaning the model predicted the class correctly for 72% of the samples compare with the real dataset.

### 4.5. Performance under the Reinforcement Learning Algorithms

**Figure 8:**
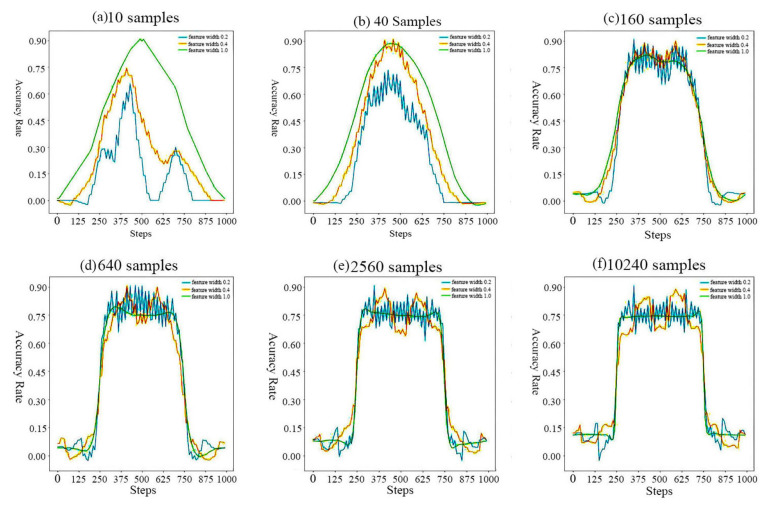
Performance under Reinforcement Learning Algorithm.

Figure 8 shows the iterative changes of the reinforcement learning algorithm under different number of samples. The three lines from top to bottom are: feature with 0.2; feature with 0.4; feature with 1.0. When we use 10,240 samples, the accuracy of the reinforcement learning algorithm model is greater than the other number of samples. So we can know the number of samples the accuracy of the prediction is higher.

Given new data points, category results are obtained by placing input dataset into the reinforcement Learning algorithm model. In order to obtain the accuracy of the reinforcement learning algorithm, we compare the predicted results of the marked dataset with those of the reinforcement learning algorithm model. We see that reinforcement learning model is about 94% accuracy, meaning the model predicted the class correctly for 94% of the samples compare with the real dataset.

### 4.6. Performance Comparison of Different Algorithms

**Figure 9:**
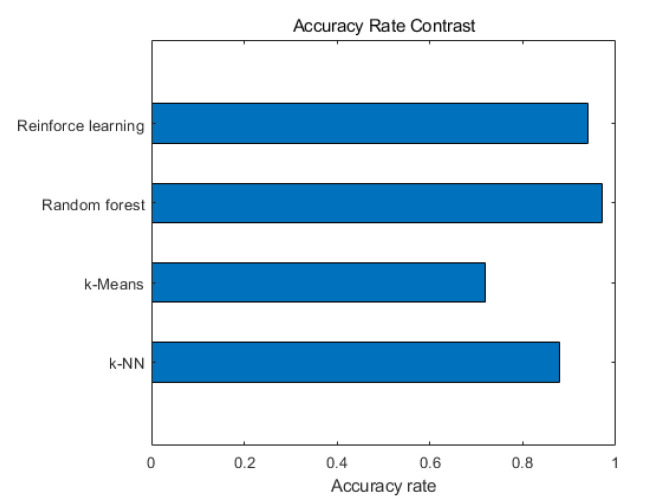
Comparing the accuracy rate of four algorithms.

**Figure 10:**
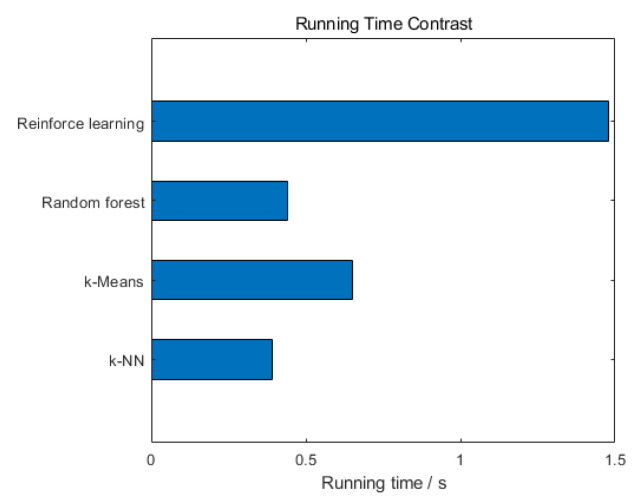
Comparing the running time of four algorithms.

We obtained the running time and accuracy of four machine learning algorithms in judging path quality from simulation experiments. Figure 9 compares the accuracy of the four algorithms. Among them, the random forest algorithm has the highest accuracy, up to 97%.The accuracy of the reinforcement learning algorithm is 94%. The accuracy of *k*-NN algorithm is 88%. The accuracy of *k*-Means algorithm is 72%. In addition, Figure 10 compares the running time of the four algorithms. The *k*-NN algorithm runs the fastest, with a running time of 0.39 s. The running time of the Random forest algorithm is 0.44 s. The running time of the *k*-Means algorithm is 0.65 s. The running time of the Reinforce learning algorithm is 1.48 s. The research results can provide references for choosing effective machine learning algorithms in MPTCP path management. If some application scenarios require high accuracy, it is recommended to use the random forest algorithm. If the running time requirements are high, the *k*-NN algorithm is recommended. However, we usually consider these two aspects together and try to find an optimal algorithm. We can see that the accuracy of the random forest algorithm far exceeds the others, indicating that the least errors will occur when judging the quality of the path. Moreover, the random forest algorithm has better performance in terms of running time. With the development of science and technology, the computing performance of many hardware devices is constantly improving, and the running speed is getting faster and faster. Therefore, we can ignore the running time difference between the random forest algorithm and the *k*-NN algorithm. In a nutshell, we comprehensively consider the running time and accuracy of different machine learning algorithms when judging the path quality, and conclude that the random forest algorithm has the best performance.

## 5. Conclusions and Future Work

Motivated by the fact that the content-rich IoT traffic tends to be the most attractive application in the future of the Internet, and more and more IoT devices are equipped with multiple network access interfaces, nowadays, MPTCP-based multipath transmission has become a hot research topic. This paper proposes an automatic learning path selection mechanism based on MPTCP (ALPS-MPTCP) to manage the use of multiple paths based on the decisions calculated by the machine learning model. In the simulation experiment, we choose delay and packet loss rate as parameters and use the training set to train *k*-NN algorithm, random forest algorithm, *k*-Means algorithm and reinforcement learning algorithm. At the same time, we use the test dataset to evaluate and compare the performance of the four algorithms in path quality judgment. The experimental results show that the random forest algorithm has the best performance in path quality judgment considering the running time and accuracy. In future work, we will further study the application of the random forest algorithm in a vast and complex actual environment. We will continue to improve our research to achieve the five optimization goals, and try to apply the intelligent collaboration theory [34,35] and the fast learning concept [36] to optimize the path management mechanism of MPTCP in the IoT environment. We hope this work can provide some directions for researchers who apply machine learning algorithms to optimize the MPTCP path management mechanism.
